# Immunization against Multidrug-Resistant *Acinetobacter baumannii* Effectively Protects Mice in both Pneumonia and Sepsis Models

**DOI:** 10.1371/journal.pone.0100727

**Published:** 2014-06-23

**Authors:** Weiwei Huang, Yufeng Yao, Qiong Long, Xu Yang, Wenjia Sun, Cunbao Liu, Xiaomei Jin, Yang li, Xiaojie Chu, Bin Chen, Yanbing Ma

**Affiliations:** 1 Laboratory of Molecular Immunology, Institute of Medical Biology, Chinese Academy of Medical Sciences & Peking Union Medical College, Kunming, China; 2 Yunnan Key Laboratory of Vaccine Research & Development on Severe Infectious Diseases, Kunming, China; 3 Yunnan Engineering Research Center of Vaccine Research and Development on Severe Infectious Diseases, Kunming, China; 4 The Second Affiliated Hospital of Kunming Medical University; Kunming, China; Universidad Nacional de La Plata., Argentina

## Abstract

**Objective:**

*Acinetobacter baumannii* is considered the prototypical example of a multi- or pan- drug-resistant bacterium. It has been increasingly implicated as a major cause of nosocomial and community-associated infections. This study proposed to evaluate the efficacy of immunological approaches to prevent and treat *A. baumannii* infections.

**Methods:**

Mice were immunized with outer membrane vesicles (OMVs) prepared from a clinically isolated multidrug-resistant strain of *A. baumannii*. Pneumonia and sepsis models were used to evaluate the efficacy of active and passive immunization with OMVs. The probable effective mechanisms and the protective potential of clonally distinct clinical isolates were investigated *in vitro* using an opsonophagocytic assay.

**Results:**

Intramuscular immunization with OMVs rapidly produced high levels of OMV-specific IgG antibodies, and subsequent intranasal challenge with *A. baumannii* elicited mucosal IgA and IgG responses. Both active and passive immunization protected the mice from challenges with homologue bacteria in a sepsis model. Bacterial burden in bronchoalveolar lavage fluids (BALF), lung, and spleen, inflammatory cell infiltration in BALF and lung, and inflammatory cytokine accumulation in BALF was significantly suppressed in the pneumonia model by both active and passive immunization strategies. The antisera from immunized mice presented with significant opsonophagocytic activities in a dose-dependent manner against not only homologous strains but also five of the other six clonally distinct clinical isolates.

**Conclusions:**

Utilizing immunological characteristics of outer membrane proteins to elevate protective immunity and circumvent complex multidrug-resistance mechanisms might be a viable approach to effectively control *A. baumannii* infections.

## Introduction

*Acinetobacter baumannii* is a non-fermenting, Gram-negative, aerobic coccobacillus that is currently a major cause of nosocomial infections worldwide. *A. baumannii* infection leads to bacteremia, urinary tract infections, surgical site infections, and, especially, ventilator-associated pneumonia in intensive care unit patients [1]. In recent years, epidemics caused by multidrug-resistant strains of *A. baumannii* have been widely investigated and reported [1], [2]. The easy acquisition of multi- or pan-drug resistance to clinically available antimicrobial agents in this organism leads to serious therapeutic complications [3]–[6].

It is important that innovative approaches are developed to prevent and treat multi- or pan- drug-resistant infections. Immunological strategies, which may function through approaches that differ from that of antibiotics and hopefully circumvent complex multidrug-resistant mechanisms, are emerging as a viable option. Recently, several studies have shown that various vaccine candidates have an effect on controlling *A. baumannii* infections [7]–[13]. For example, inactivated whole cell [8], outer membrane complexes (OMCs) [7], and outer membrane vesicles (OMVs) of *A. baumannii* have been proven to be effective immunogens that protect mice from bacterial challenges through active or passive immunization strategies [10]. More recently, single outer membrane proteins, OmpA [12], [14], Bap [15], and Ata [11], have also been identified as effective candidate vaccines that immunologically intervene in *A. baumannii* infection.

Pneumonia and sepsis are two of the most common and severe clinical issues caused by *A. baumannii* infection [7], [8], [10], [12], [16]–[19]. The protection efficacy of vaccine immunization against *A. baumannii* has typically been evaluated using a sepsis model [7], [8], [10], [12], whereas these evaluations have seldom been performed in a model of pneumonia. The pneumonia model mimics the route and settings of clinical infection by *A. baumannii*, in which bacteria are directly in contact with the airway surface; thus, it might be important of successfully establishing both local mucosal and systematic specific immunity to produce immediate and effective protection. In the current study, we focus on the effects of OMV immunization in a pneumonia model of *A. baumannii* infection using both active and passive approaches. Moreover, a sepsis model was used to confirm the effectiveness of the vaccine by observing survival rates. The data presented here facilitated an overall evaluation of the protection produced by the immunological approaches against infection by drug-resistant *A. baumannii* and furthered our knowledge of *A. baumannii* infection and immunization.

## Materials and Methods

### Ethics Statement

The animal experimental procedures were approved by the Ethics Committee of Animal Care and Welfare, Institute of Medical Biology, CAMS (Permit Number: SYXK (dian) 2010–0007), in accordance with the animal ethics guidelines of the Chinese National Health and Medical Research Council (NHMRC) and the Office of Laboratory Animal Management of Yunnan Province, China. All efforts were made to minimize animal suffering.

All participants submitted a signed informed consent form to participate in the study. The protocol complied with the Helsinki Declaration and was approved by the Institutional Review Boards of the Institute of Medical Biology, Chinese Academy of Medical Sciences & Peking Union Medical College.

### Bacterial strains and mice

*A. baumannii* strains 1 to 7 (Ab1 to Ab7) were isolated from intensive care unit (ICU) patients hospitalized at the affiliated hospitals of Kunming Medical University (Kunming, China). Ab1, Ab4, Ab5, Ab6, and Ab7 were isolated from sputum, and Ab2 and Ab3 were isolated from peritoneal fluid drainage and blood, respectively. All strains are pan-drug resistant against a panel of 17 known antibiotics [20]. These strains were confirmed through DNA sequencing analysis of the intergenic spacer (ITS) between 16 S and 23 S rRNA genes based on the methods described by Chang et al. [21] The strains were grown in Luria-Bertani (LB) medium under the selection pressure of ampicillin and kanamycin antibiotics. Female ICR mice (6–8 weeks of age) were raised and maintained in the Central Animal Care Services of the Institute of Medical Biology, Chinese Academy of Medical Sciences (CAMS) & Peking Union Medical College (PUMC) (Kunming, China) under specific pathogen-free (SPF) conditions.

### OMV preparation

A single Ab1 colony was inoculated and cultured in LB medium at 37°C, overnight. The bacterial culture was centrifuged at 14,000×g for 15 min, and the supernatant was filtered through a 0.22-µm membrane (Millipore, Merck, Darmstadt, Germany). The filtered fraction was concentrated by ultra-filtration with a 500,000 nominal molecular weight cut off (500,000 NMWC) column (QuixStand Benchtop System, GE Healthcare, Piscataway, NJ, USA). The concentrate was ultra-centrifuged at 200,000×g for 2 h at 4°C. The vesicle pellets were resuspended in phosphate buffered saline (PBS; 0.02 mol/L phosphate buffer with 0.15 mol/L NaCl, pH 7.4) and then filtered through a 0.22-µm membrane [10]. The absence of viable bacteria in the OMV preparations was determined by spreading aliquots on agar plates to test for bacterial growth. OMVs were quantified using Bradford reagent according to the manufacturer's instructions.

### Electron microscopy

The OMV sample was fixed with 2.5% cold glutaraldehyde in 0.2 M sodium cacodylate buffer (pH 7.4) for 2 h at 4°C and postfixed with 1% osmium tetroxide in 0.1 M sodium cacodylate buffer (pH 7.4) for 1 h at 4°C; then, the sample was observed and imaged using a transmission electron microscope (Hitachi, Tokyo, Japan) at 80 kV.

### Mouse immunizations

A preliminary study was performed using different vaccine doses (5, 25, 50 and 100 µg/ml) to immunize the mice, and the results showed that 50 µg/ml is most likely the optimal vaccination dose (data not shown). In this study, the vaccine was prepared by mixing the OMVs (isolated from Ab1) with an equal volume of Alum adjuvant (Thermo Scientific, Rockford, IL, USA) to a final concentration of 50 µg/mL OMVs and 2 mg/mL Alum. Then, each mouse was immunized intramuscularly with 200 µL of the vaccine three times, on days 0, 15, and 36. For studies with active immunization, an additional group of mice were injected with a mixture of PBS and adjuvant to serve as the control. For all active immunization studies, the challenge with homologous strains of *A. baumannii* occurred 11 days after the last immunization with OMVs (day 47). In addition, for the passive immunization studies, either 100 µL of naive serum or antiserum collected on day 52 from the vaccinated mice was administered intravenously 1 h prior to the bacterial challenge.

### Western blot assays

The outer membrane complex (OMCs) and whole cell (WC) proteins were isolated from Ab1 as described previously [7], [8]. OMVs, OMCs, and WC samples were quantified using the Bradford reagent, loaded and separated on a 15% SDS-PAGE, and transferred to a polyvinylidene difluoride (PVDF) membrane (Pall Corporation, East Hills, NY, USA). The membrane was blocked with 5% milk powder and then incubated with sera collected from experimental mice at a dilution of 1∶3000. The sera collected on day 52 from the OMV-immunized or adjuvant only-treated mice were pooled for the assays. Horseradish peroxidase (HRP)-conjugated rabbit anti-mouse IgG (Life Technologies, Gaithersburg, MD, USA) was used as a secondary antibody. The blot was developed with enhanced chemiluminescence (ECL) substrate according to the manufacturer's instructions (Pierce, Rockford, IL, USA). The WC samples from seven separately collected clinical isolates were also analyzed with the antisera.

### Pneumonia and sepsis models

A sepsis model was established as described previously, with minor modifications [12]. Briefly, Ab1 under exponential growth conditions was collected and adjusted to the designated concentrations with PBS according to optical density 600 (OD_600_) values based on a previously determined relation curve between optical density and colony forming units (CFUs). Inocula were prepared by mixing the bacterial suspension with 10% porcine mucin (w/v; Sigma-Aldrich, St Louis, MO, USA). Mice were injected intraperitoneally with 0.5 mL of the inocula, and the actual numbers of CFU reflecting injected bacterial loads were determined by plating sequential dilutions of the inocula on LB plates.

The pneumonia model was established as described previously, with minor modifications [22]. In brief, the mice were anesthetized with inhalation isoflurane (Baxter Healthcare Corporation, Glendale, CA, USA), and 50 µL of live Ab1 (10^8^ CFU/mL) in PBS was administered intranasally (i.n.). Control mice were administered PBS.

### Sample collection

At the conclusion of the experiment, the mice were anesthetized and blood samples were obtained by cardiac puncture. Bronchoalveolar lavage fluid (BALF) was collected with 1 mL of ice cold PBS, flushing slowly three times. After centrifugation, the supernatants were kept for antibody and cytokine measurements. Cells were resuspended with PBS and counted using a hemocytometer, and differential analysis of inflammatory cells was performed on cytospin preparations (Cytospin, Shandon, Runcorn, UK) stained with HEMA 3 (Fisher Diagnostics, Pittsburg, PA, USA). The left lung was removed and immersed immediately in 10% neutral buffered formalin for histological analysis. The right lung and spleen were removed aseptically from the mice, weighed, added to 1 mL of ice-cold PBS, and homogenized using a Tissue Tearor (Biospec Products, Racine, WI, USA) to determine the bacterial burden.

### Cytokine measurement by ELISA

The concentrations of cytokines (IL-1β, IL-13, IL-33, IFNγ, and TNFα) in BALF were detected using ELISA, which was performed using paired capture and biotinylated detection antibodies purchased from eBioscience, Inc. (San Diego, CA, USA) according to the supplier's instructions.

### Bacterial burden

The bacterial burden was determined in BALF and lung and spleen homogenates by plating 10-fold dilutions on LB plates with double antibiotic resistance and incubating at 37°C overnight. The number of colony forming units was counted, and the results are expressed as the numbers of CFU/g tissue or the numbers of CFU/mL BALF.

### Histological analysis

Lung tissues were embedded in paraffin and serially sectioned (5 µm) sagittally. Specimens were stained with hematoxylin and eosin to examine peribronchial and perivascular accumulation of inflammatory cells.

### Antibody measurement by ELISA

At the designated time points during the experiment, blood samples were collected by sphenous sampling from the limb. At the end of experiment, the mice were anaesthetized, and blood samples were obtained by cardiac puncture. Measurements of OMVs-specific IgA and IgG antibodies were performed successively by coating microplates with previously prepared OMVs (2 µg/mL), adding serum or BALF samples, incubating with alkaline phosphatase-conjugated goat anti-mouse IgA or IgG (Life Technologies, Gaithersburg, MD, USA) and adding substrate tablets (Sigma-Aldrich, St Louis, MO, USA) to develop the reaction. The serum levels of OMV-specific IgG_1_ and IgG_2a_ were also measured. Briefly, after sample incubation, either biotinylated goat anti-mouse IgG_1_ or IgG_2a_ (Life Technologies, Gaithersburg, MD, USA) was used as a secondary antibody, and the reaction was developed using an avidin–phosphatase system.

### Opsonophagocytic assay

The opsonophagocytic killing assay was performed as previously described with modifications [12]. Murine macrophage RAW264.7 cells were cultured in DMEM/HIGH Glucose (HyClone, Logan, UT, USA) with 10% fetal bovine serum (FBS). The cells were activated with a 3-day exposures to 100 nM phorbol 12-myristate 13-acetate (PMA; Sigma-Aldrich, St Louis, MO, USA), harvested by scraping with cell scrapers (Thermo Scientific, Rockford, IL, USA), and added to the microplates at a 40∶1 ratio of macrophages (∼2×106 cells/mL) to Ab 1 to Ab 7 (∼5×104 CFU/mL). The pooled OMV antisera were diluted sequentially at 1∶1, 1∶10, 1∶100, 1∶1000, and 1∶10000 with culture medium. In addition to a pooled naive serum, a pooled irrelevant antigen-immunized serum was used as a control to confirm that there were no differences between the normal and the OMV-irrelevant immunization sera with respect to the killing effect. The irrelevant antigen is a recombinant fusion protein that consists of glutathione S-transferase (GST) and enhanced green florescence protein (EGFP), which was previously prepared in our lab using gene recombinant technology. The sera were subjected either to no heat or to heat treatment at 56°C for 30 min to inactivate the complement components. The test sera, bacteria, and macrophages were mixed and then incubated for 1 hour with gentle shaking, and the mixtures were diluted and plated for bacterial counting. The number of non-phagocytosed bacteria was determined, which reflects the number of bacteria phagocytosed via another pathway.

### Statistical analyses

All statistical analyses were performed using Prism 5.0 (GraphPad Prism, GraphPad Software, Inc., San Diego, CA, USA). Survival was compared using the non-parametric log-rank test. The bacterial burdens were compared with an unpaired Student's t test. One-way ANOVA with Tukey's multiple comparison test was applied for the analyses of cytokine, IgG_1_, and IgG_2a_ levels. Differences were considered significant if the *p* value was <0.05. All graphed values represent the mean, and the error bars represent the standard error.

## Results

### Immunization with OMVs produces a high level of persistent IgG responses to *A. baumannii*

**Figure 1:**
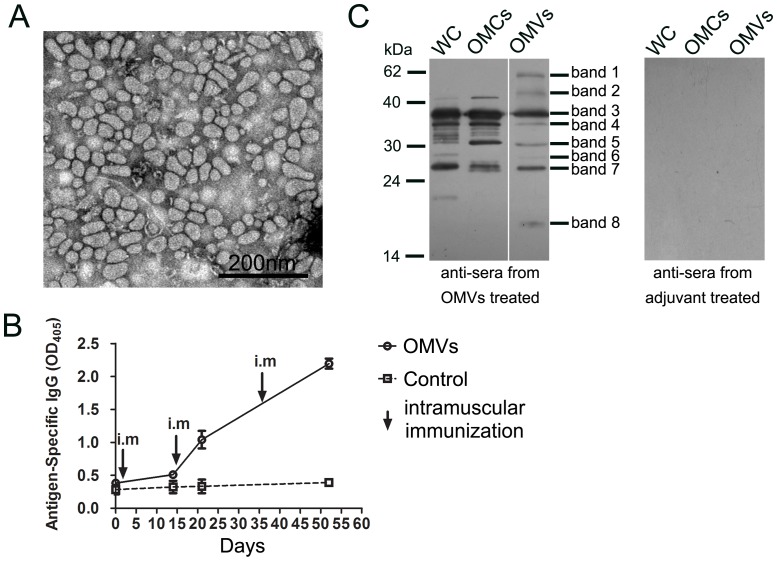
Immunization with OMVs produced high, persistent IgG response to *A. *baumannii** (A) OMVs were prepared from the supernatant of bacterial culture and imaged using a transmission electron microscope; bar, 2000 nm. (B) The kinetic responses of OMV-specific IgGs were measured by ELISA. (C) Western blots treated with the antisera collected from the OMV-vaccinated mice displayed specific reactions to *A. baumannii* proteins, whereas blots treated with sera from adjuvant only-treated mice showed no reactive bands. The sera collected on day 52 were pooled and used to generate these blots. WC, whole cell proteins; OMCs, outer membrane complexes; OMVs, outer membrane vesicles.

OMVs were prepared from the supernatant of bacterial culture after centrifugation, observed for morphology, and imaged using a transmission electron microscope (Figure 1A). The size of the vesicles ranged from 20 to 100 nm, which was similar to that reported previously [10].

Each mouse was immunized intramuscularly three times on days 0, 15, and 36 with OMVs prepared from strain Ab1, and the challenge with the homologue strain *A. baumannii* occurred 11 days after the last immunization. To demonstrate the dynamic features of the IgG responses after OMV immunization (n = 6), mouse sera were collected on days 0, 14, 21, and 52, and ELISA was used to measure the levels of OMV-specific IgGs (Figure 1B). IgG levels were only slightly elevated 14 days after the primary immunization. However, the levels increased after the booster immunizations, and the titers reached 16,000 on day 14 (7 days after the first booster) and 64,000 on day 36 (21 days after the second booster). The control mice did not show any OMV-specific IgG responses.

### Characterization of immunogenic proteins in OMVs

Proteomic studies on OMVs from *A. baumannii* have indicated that OMVs contain proteins from the outer membrane, the inner membrane, the periplasm, and the cytoplasm [23], [24], whereas a recent study based on cryo-electron tomography showed that OMVs at the distal ends of early log-phase bacteria did not contain cytoplasmic proteins but only outer membrane and periplasmic proteins. However, inner-outer membrane vesicles are produced by *A. baumannii* in the stationary phase, and they also contain cytoplasmic proteins [25]. To establish the dominant antigen components in the OMV preparation in the current study and determine their possible cellular distribution, OMVs, OMCs, and WC proteins were used for western blot analyses with the antisera collected from vaccinated mice or adjuvant only-treated mice on day 52 (Figure 1C). There were no reactive bands when the sera from adjuvant only-treated mice were used (Figure 1C, right panel). Antisera from the vaccinated mice showed that there were 8 major specifically reactive bands observed in the OMVs (Figure 1C, left panel). Bands 3, 4, 5, and 7 were observed in all samples, indicating that they are membrane proteins because they were clearly detected in both OMC and OMV samples. Band 3, with a molecular weight of approximately 38 kDa, has been reported to be OmpA and used as a vaccine candidate [12], [14], and it was the most dominant component in all three samples. Bands 1, 2, and 8 were only observed in the OMVs, strongly indicating that they were highly immunogenic, relatively enriched in OMVs, and most likely periplasmic proteins, considering that no similar bands appeared in OMCs. The other bands appeared only in WC or/and OMCs and were more likely to be non-membrane proteins because of their low levels in OMVs. In summary, the Western blot results showed that vaccination with OMVs elicited antibodies against multiple bacterial antigens, the majority of which are most likely located on the bacterial outer membrane.

### Immunization with OMVs increases the survival of mice in a sepsis model

**Figure 2:**
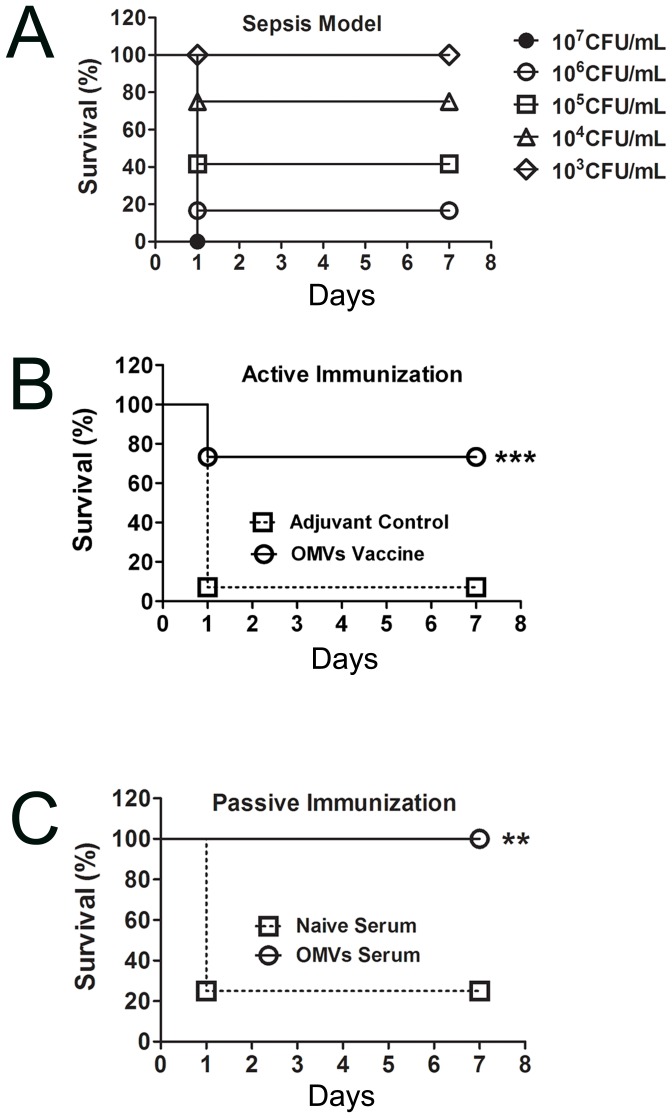
Immunization with OMVs increased mouse survival in a sepsis model. (A) The survival of mice that were challenged with different numbers of colony forming units of *A. baumannii* was observed; (B) Mice that were actively vaccinated with OMVs had a higher survival rate than the control mice in response to the *A. baumannii* challenge 11 days after the last immunization; (C) Mice that passively received antiserum were completely protected from *A. baumannii* infection; there were no deaths in the subsequent 2 weeks. Mice were injected intraperitoneally with 50 µL of live Ab1 (1.1×10^6^ CFU/mL) mixed (1∶1, v/v) with 10% porcine mucin (w/v) in both the active and passive immunization approaches. The *p* value was determined by the non-parametric log-rank test. **, *p*<0.01, ***, *p*<0.001; n = 12 to 15 mice per group.

First, the effectiveness of the vaccine was confirmed in a sepsis model. To establish the sepsis model, mice (n = 12/group) were injected intraperitoneally with 0.5 mL of different doses of *A. baumannii*: 1×10^3^ CFU/mL; 1×10^4^ CFU/mL; 1×10^5^ CFU/mL; 1×10^6^ CFU/mL; and 1×10^7^ CFU/mL. The survival rate was calculated continuously over the next 7 days (Figure 2A). Correspondingly, the survival rates in the experimental groups were 100, 75, 41.67, 16.67, and 0%, respectively, and all mouse deaths occurred within 24 h. The bacterial dose of 5.5×10^5^ CFU/mouse was used as a challenge dose 11 days after the last immunization to assess the efficacy of the active and passive immunization approaches. The results showed that the mice vaccinated with OMVs (n = 15) had a higher survival rate than the adjuvant control mice (n = 14), with 73.3% survival versus 7.14% (*p*<0.001, Figure 2B). In addition, the mice that were administered passive treatment with antisera were completely protected from *A. baumannii* infection, whereas only ∼20% of the mice survived in the control group after being administered naive serum (*p*<0.01, non-parametric log-rank test, n = 12, Figure 2C). As a brief summary, both active and passive immunization with OMVs protected the mice from a challenge with the *A. baumannii* strain from which the OMVs were derived.

### Immunization with OMVs reduces bacterial burden in a pneumonia model

**Figure 3:**
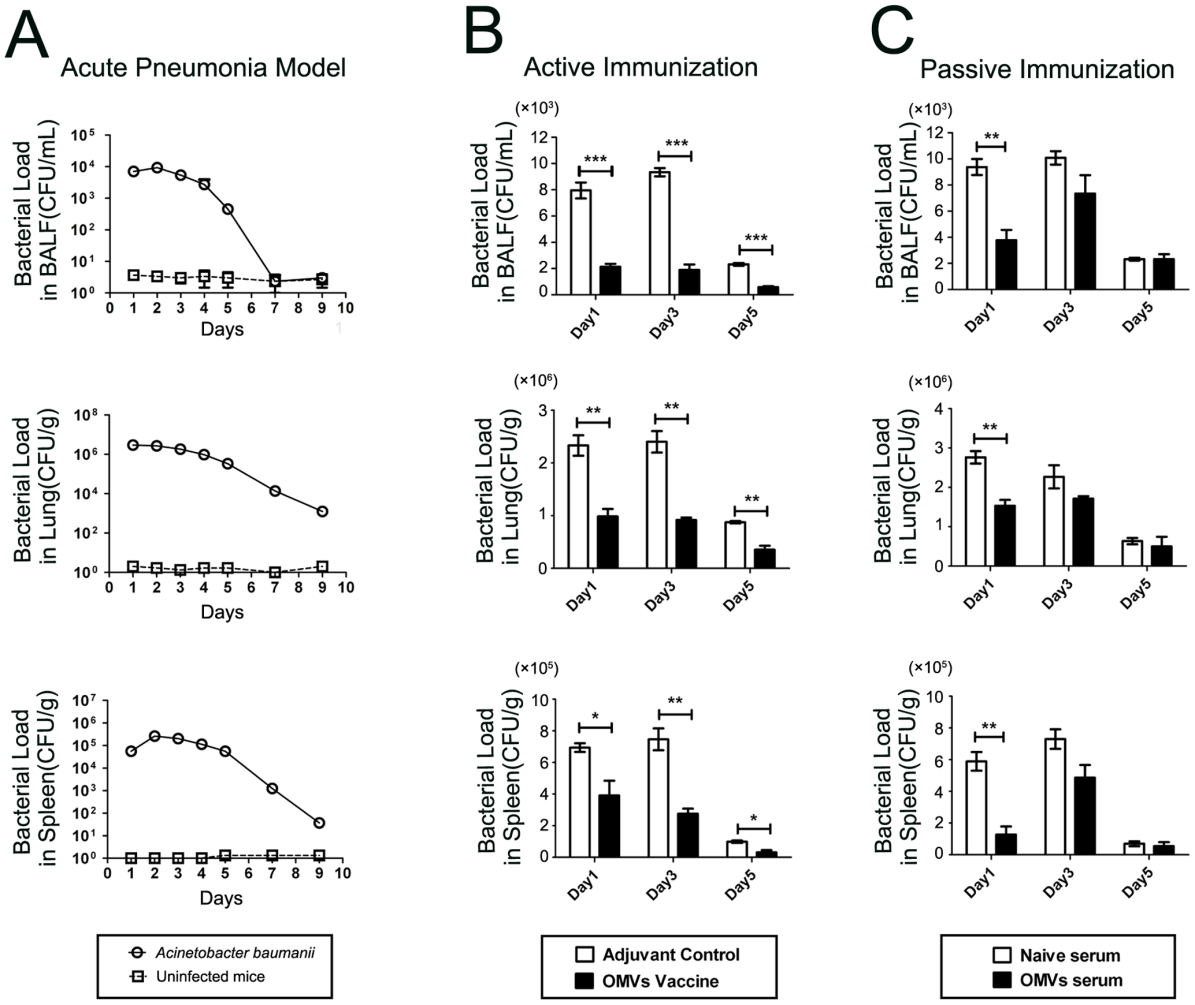
Immunization with OMVs reduced bacterial burden in a pneumonia model. (A) The bacterial loads in BALF, lung and spleen were detected continuously for 9 days in the pneumonia model. The *A. baumannii* group was challenged with *A. baumannii*, and the control group received no challenge; (B) The mice that were actively vaccinated with OMVs showed a significant reduction in bacterial loads; (C) The mice that received passive immunization with the antisera were completely protected from *A. baumannii* infection. The *p* value was determined by an unpaired Student's t test. *, *p*<0.05, **, *p*<0.01, ***, *p*<0.001; n = 12 mice per group.

Mice were intranasally administered 50 µL of live Ab1 (10^8^ CFU/mL). No deaths occurred in the following 2 weeks. The kinetics of the bacterial burden in major tissues were investigated to determine bacterial clearance to establish an optimal time point for other measurements. The bacterial burden in BALF, lung, and spleen was detected continuously in the mice challenged with the *A. baumannii* homologs (n = 12) and in the control mice administered PBS instead of *A. baumannii* (n = 12; Figure 3A). The bacterial loads decreased from day 3 and persisted for at least 5 days in the BALF and 9 days in the lung and spleen. To determine the protective effects of active and passive immunization strategies, the bacterial burden was analyzed on days 1, 3, and 5 after the *A. baumannii* challenge. With regard to active immunization, the results showed that the OMV vaccine (n = 12) significantly reduced the bacterial loads in the BALF, lung, and spleen compared with the adjuvant controls (n = 12). The mean of the CFU values on days 1, 3, and 5 were as follows: BALF − 5815, 7460, and 1730.6, respectively; lung − 1,346,000, 1,485,000, and 520,000, respectively; and spleen − 303,000, 471,000, and 67,900, respectively. The protective effect lasted for at least 5 days (Figure 3B). In addition, the mice that were administered the antisera one hour prior to the bacterial challenge showed a significantly reduced bacterial burden on day 1. However, there was a trend toward a reduced bacterial burden on day 3, and no difference was observed on day 5 between the immunized mice (n = 12) and the control mice (with naive serum, n = 12). The CFU values on day 1 were 5603, 1,260,000, and 462,000 in the BALF, lung, and spleen, respectively. A significant reduction in the bacterial burden by passive immunization was observed only on day 1 (Figure 3C).

### Immunization with OMVs reduces pulmonary infiltration of inflammatory cells in a pneumonia model

**Figure 4:**
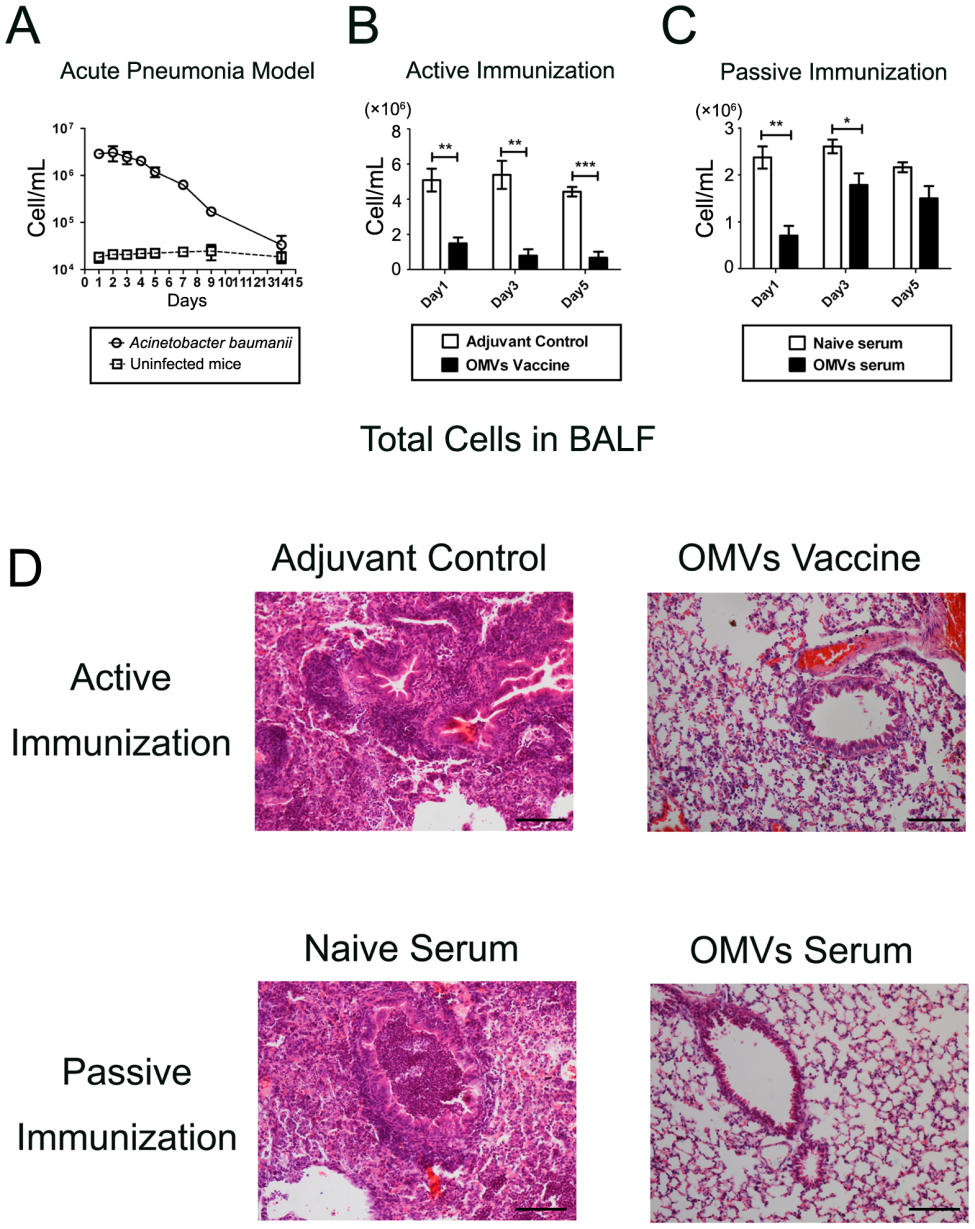
Immunization with OMVs suppressed pulmonary inflammatory cell infiltration in a pneumonia model. (A) Dynamic accumulation of inflammatory cells in BALF in the pneumonia model; the *A. baumannii* group was challenged with live *A. baumannii* (Ab1), and the control group received no challenge; (B) and (C) Active or passive immunization significantly suppressed inflammatory cell accumulation in BALF; (D) Representative photomicrographs of lung histology showed that both active and passive immunization ameliorated inflammatory cell infiltration in the lung 3 days after the *A. baumannii* challenge. The sections were stained with hematoxylin and eosin. Bar, 100 µm. The *p* value was determined by an unpaired Student's t test. *, *p*<0.05, **, *p*<0.01, ***, *p*<0.001; n = 12 mice per group.

In the pneumonia model (n = 12/group), the accumulation of immune cells dominated by neutrophils was significantly increased in the BALF after bacterial challenge, which continued until day 14 (Figure 4A). Immunization with OMVs, including both active and passive approaches, significantly decreased inflammatory cell accumulation in the BALF, which corresponded to a reduction in the bacterial burden. Active immunization presented a significant reduction in inflammatory cell accumulation on all the investigated days after the *A. baumannii* challenge (n = 12/group; Figure 4B). However, passive immunization showed a significant difference between the vaccinated mice (n = 12) and the control mice (with naive serum, n = 12) on days 1 and 3 only. There was only a trend toward reduction on day 5 (Figure 4C). We also examined the histopathological changes in the lungs. The *A. baumannii* challenge caused significant bronchial, peribronchial, and perivascular infiltration of inflammatory cells. Both active and passive immunization significantly reduced inflammatory cell infiltration in the lungs of the immunized mice compared with the control mice (3 days after the *A. baumannii* challenge; Figure 4 D).

These results were consistent with the significant decrease in the inflammatory cell accumulation in the BALF, indicating that pulmonary inflammation caused by the *A. baumannii* infection was significantly suppressed by the immunization with OMVs.

### Immunization with OMVs ameliorates the cytokine storm in BALF in a pneumonia model

**Figure 5:**
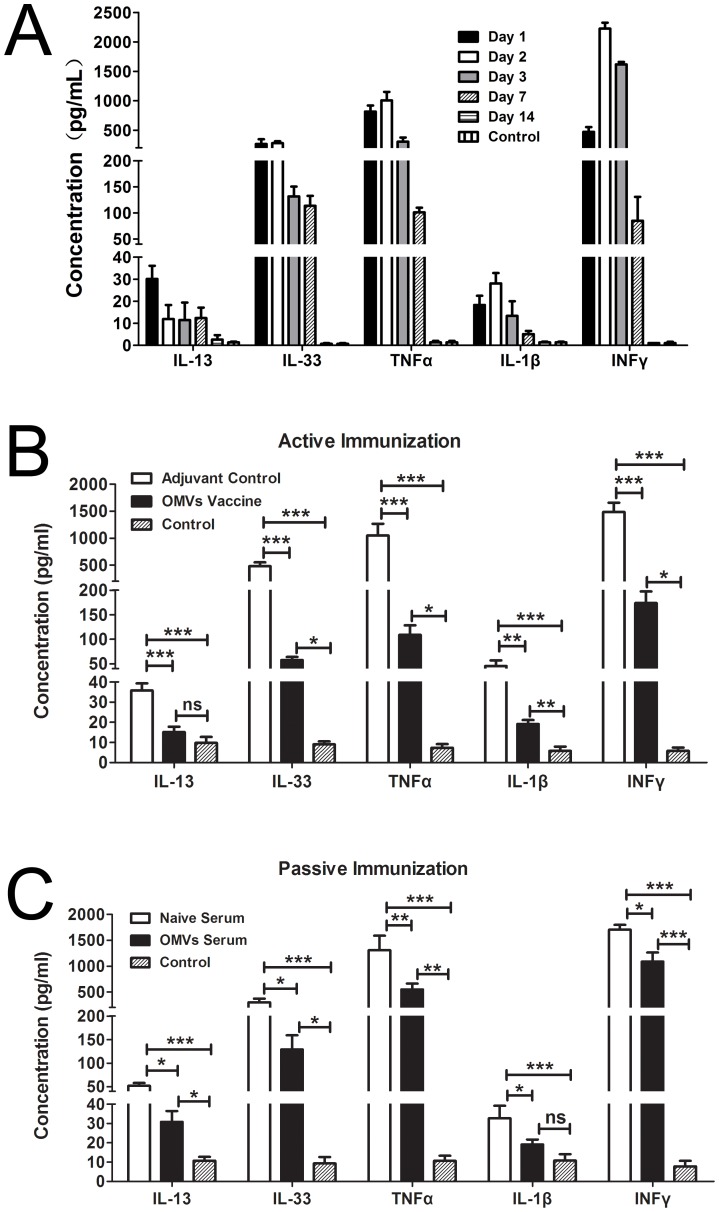
Immunization with OMVs reduced cytokine accumulation in BALF in a pneumonia model. (A) Dynamic accumulation of inflammatory cytokines in BALF in the pneumonia model; (B) and (C) Both active and passive immunization ameliorated inflammatory cytokine accumulation in BALF. The levels of cytokines were measured 3 days after intranasal challenge with live *A. baumannii* using ELISA. The control represents samples from normal mice. The *p* value was determined by one-way ANOVA with Tukey's multiple comparison test. *, *p*<0.05, **, *p*<0.01; ***, *p*<0.001; n = 12 mice per group.

The intranasal challenge with *A. baumannii* caused a cytokine storm in the BALF. The levels of IL-13, IL-33, IFNγ, TNFα, and IL-1β were measured on days 1, 2, 3, 7, and 14 (n = 12/group). All cytokines increased dramatically in the infected mice compared with the controls on all investigated days, except for day 14, when the cytokine levels returned to baseline. The increase in IFNγ, TNFα, and IL-1β in this context has been previously reported [7]. Interestingly, the Th2 cytokines IL-13 and IL-33 were also found to be elevated. Cytokine levels decreased gradually from day 3, reaching baseline levels on day 14 (Figure 5A). Both active and passive immunization (n = 12/group) significantly lowered the levels of cytokines in the vaccinated mice compared with the control mice 3 days after the *A. baumannii* challenge (Figure 5B&C). Corresponding to the bacterial burden and inflammatory cell infiltration, the cytokine accumulation in BALF was suppressed more significantly by active immunization than by passive immunization between the adjuvant control and OMV vaccine.

### IgA and IgG responses in serum and BALF after immunization and bacterial challenge in a pneumonia model

**Figure 6:**
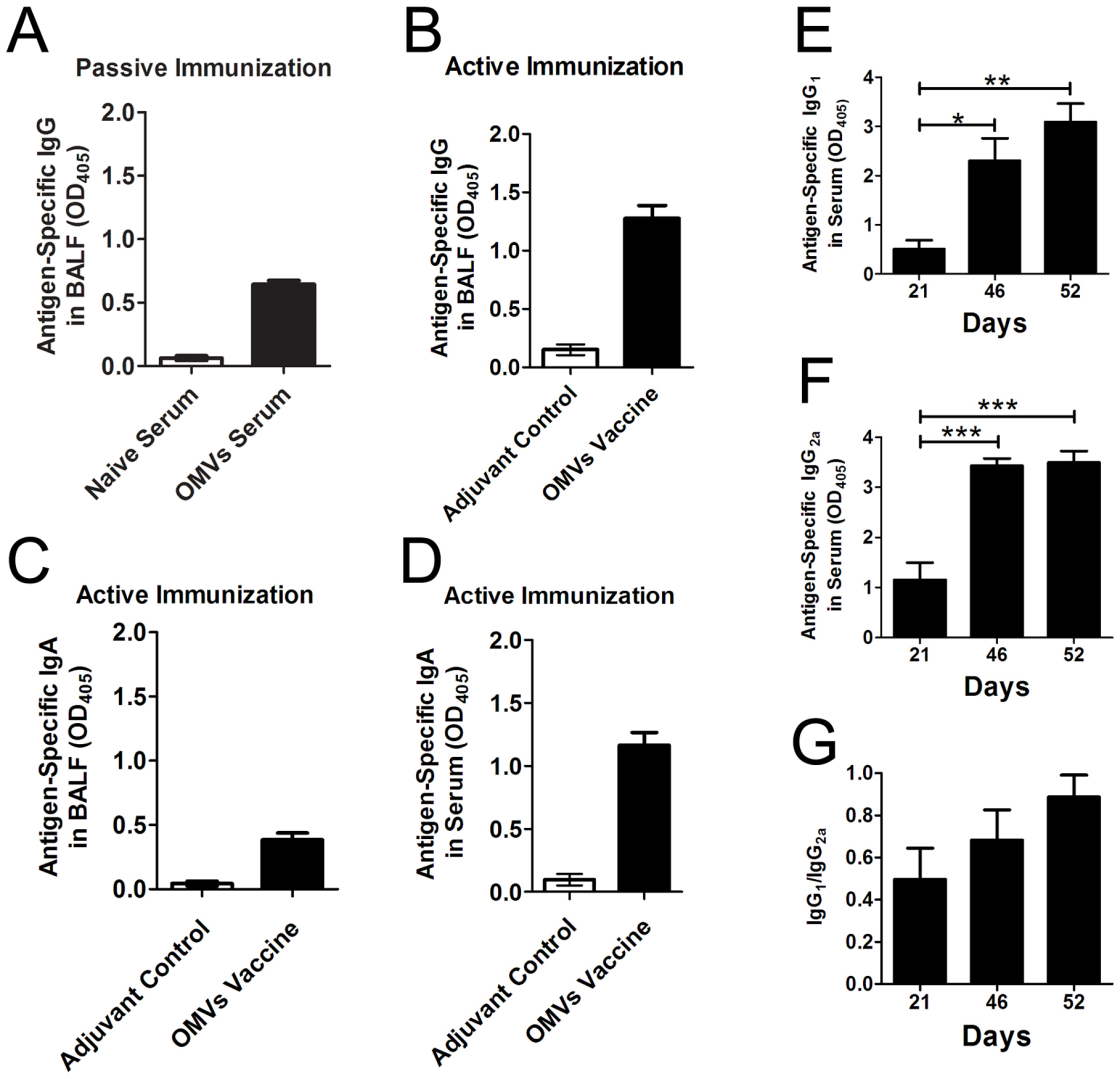
IgA and IgG responses in serum and BALF after immunization and bacterial challenge. (A) OMV-specific IgGs in BALF were detected in mice that were passively immunized with antiserum; (B) OMV-specific IgGs in BALF were detected in mice receiving active immunization; (C) and (D) OMV-specific IgAs were detected in BALF and serum, respectively. The samples were collected 5 days after the bacterial challenge. (E), (F), and (G). Immunization with OMVs elicited Th1-like antibody responses. OMV-specific IgG_1_ and IgG_2a_ levels were measured using ELISA, and the ratio of IgG_1_/IgG_2a_ was calculated for each mouse. The bacterial challenge occurred 11 days after the last immunization. The *p* value was determined by one-way ANOVA with Tukey's multiple comparison test. *, *p*<0.05, **, *p*<0.01, ***, *p*<0.001; n = 12 mice per group.

The levels of antigen-specific IgA and IgG were measured in the serum and BALF samples using ELISA (n = 12/group). The samples were collected on day 52 (day 5 after the challenge), which was the end of this experiment. The results showed that OMV-specific IgG was clearly detectable in BALF after passive intravenous immunization with the antisera from vaccinated mice (Figure 6A), and active immunization with OMVs showed higher IgG levels compared with passive immunization (Figure 6B). Interestingly, following the intranasal *A. baumannii* challenge, high levels of OMV-specific IgAs were dramatically induced in both the serum and BALF in those mice that were intramuscularly immunized with the OMVs (Figure 6C&D). The control mice had no detectable OMVs-specific IgA or IgG antibodies.

To determine the Th1 or Th2 response profile to the OMV immunization and *A. baumannii* infection, the OMV-specific IgG_1_ and IgG_2a_ levels were determined using ELISA. A 1∶500 dilution for the serum samples was used for both the IgG_1_ and IgG_2a_ measurements. The results showed that the OMV immunization elicited strong IgG_2a_ responses in addition to the induction of IgG_1_, and subsequent pulmonary infection produced a response profile of the antibody subtypes similar to that induced by the OMV immunization (Figure 6E&F). The IgG_1_/IgG_2a_ ratios indicated a balanced response of the IgG subtypes evoked by either OMV immunization or *A. baumannii* infection (Figure 6G).

### The antisera from immunized mice showed a remarkable opsonophagocytic killing activity of homolog strains

**Figure 7:**
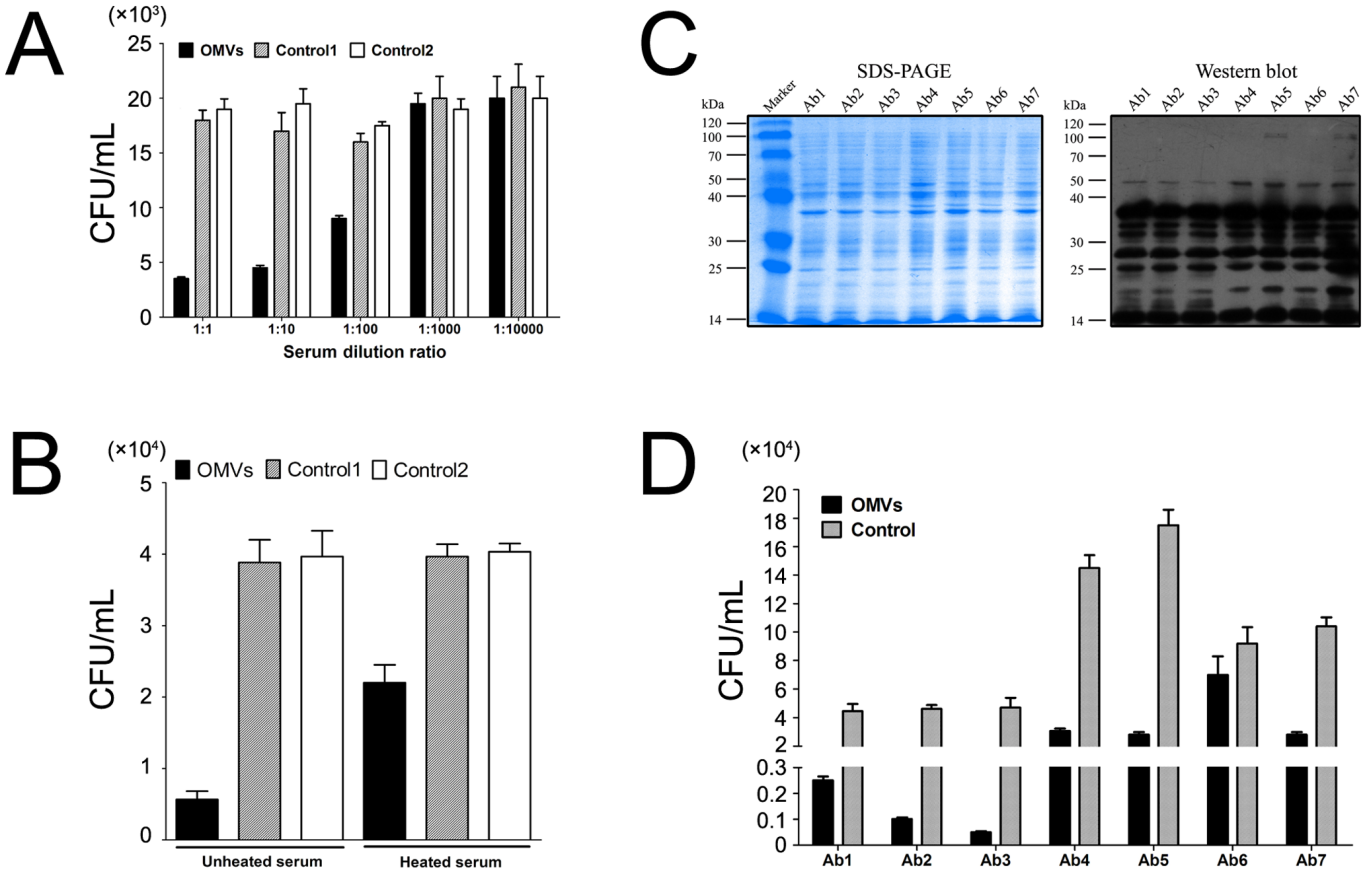
Antisera from immunized mice showed remarkable opsonophagocytic killing of clonally distinct clinical isolates. (A) The antisera from the vaccinated mice presented distinct opsonophagocytic activity in a dose-dependent manner against the homolog strain. Control 1 is a naive serum and control 2 is an irrelevant antigen GFP-immunized serum with Alum as the adjuvant. (B) The complement components were also involved in the antibody-mediated opsonic phagocytosis. The sera were subjected to no heat or heat treatment at 56°C for 30 min to inactivate the complement components. (C) SDS-PAGE and Western blot assays showed similar patterns for 7 clonally distinct clinical isolates of *A. baumannii*. The antisera collected from the vaccinated mice were pooled and used in the western blot assay. The images are representative of all the experimental replicates. (D) The antisera from the vaccinated mice remarkably reduced the survival of six of the seven clinical isolates. The mouse macrophage RAW264.7 cell line was used for the opsonophagocytic assay. The results presented here are from one of three separate repeated experiments that showed highly similar results.

The pooled antisera mediated a distinct phagocytic response to the *A. baumannii* homologs in RAW264.7 macrophages in a dose-dependent manner compared with the controls (Figure 7A). Complement components were also involved in the opsonic phagocytosis because the complement-inactivated serum showed a reduced killing effect against bacteria (Figure 7B). Two control sera were used in these experiments, and the results showed that there were no differences in the plating CFUs between control 1 (naive serum) and control 2 (irrelevant antigen GST/GFP-immunized serum with Alum as adjuvant). The results presented here are from one of three separate experiments that showed highly similar results.

### The antisera from immunized mice showed a remarkable opsonophagocytic killing activity of clonally distinct clinical isolates

To investigate whether the antisera had the potential to protect against non-homologue strain challenges, seven clonally distinct clinical isolates were used for the killing test. The whole cell proteins of the seven isolates were analyzed with SDS-PAGE, and Western blot assays were performed with the antisera from the vaccinated mice (Figure 7C). The results showed that the antisera reacted to the whole cell preparations of the seven isolates in almost identical patterns, with similar specifically reactive bands. The subsequent opsonophagocytic assay showed that the pooled antisera from the homolog strains for vaccine preparation remarkably reduced the survival of six of the seven isolates, with no apparent effect on one isolate (Figure 7D). The results presented here are from one of three separate experiments that showed highly similar results.

## Discussion

Infection with *A. baumannii* causes severe issues in critically ill patients, mainly due to its inherently complicated drug-resistant mechanisms and rapid acquisition of multi- or even pan- resistance. Furthermore, the development of new antimicrobial reagents has been challenging. Therefore, a distinct approach utilizing the immunological characteristics of the outer membrane of *A. baumannii* might be a viable alternative.

Recently, inactivated whole cells, OMCs, and OMVs of *A. baumannii* have been developed as effective vaccine candidates. The data showed that both active and passive immunization are able to protect against challenges not only from homologue strains but also clonally distinct clinical isolates [7], [8], [10]. More recently, individual recombinant proteins, including OmpA, Bap, and Ata, and the K1 capsular polysaccharide have been produced and identified as potential immunogens to fight against infection by *A. baumannii* [11]–[13], [15]. Given that inactivated whole cells contain unnecessary components that may disturb the induction of specific immune responses to effective antigens and may have safety issues and that OMCs require complex purification procedures and most likely the use of a strong adjuvant [7], [8], OMVs have the advantages of better safety and stronger immunogenicity endowed by the vesicle structure with sizes of 20-100 nm [10]. An OMV vaccine against Neisseria meningitidis type B has been developed and applied successfully in humans in New Zealand, indicating an acceptable safety and effectiveness profile of the OMV vaccination strategy [26]–[28]. Furthermore, individual outer membrane proteins may have to face the serious challenge of the variable antigenicity of variant clinically isolated strains and weak immunogenicity. In contrast, OMVs have self-adjuvant effects and elicit an antibody response against multiple outer member proteins, thereby providing broad and effective protection. Taken together, OMVs appear to be more attractive and promising than other vaccine candidates.

The ICR mouse is a general-purpose model used for studies in a wide range of fields, including toxicity, pharmacology, drug efficacy, and immunology. There have been many previously published vaccine studies on ICR mice [29]–[32]. ICR mice are outbred mice with genetic functional diversity. Compared with inbred strains, one of the perceived advantages of using outbred strains is that they more accurately mimic human genetics; the disadvantage is that the results may be substantially more difficult to reproduce. An increased number of animals is often required to overcome this disadvantage. Considering that OMV immunization has been proven to be effective at preventing A. baumannii infection in sepsis or pneumonia models in previous reports using inbred mice, such as C57BL/6 [10] or C3H/HeN [9], in the current study, we used outbred ICR mice to further our knowledge of the potency of OMV immunization in both sepsis and pneumonia models with the consideration of possible genetic diversity. Although it may not reflect clinical settings, this approach may provide additional useful information.

Using a sepsis model, the current study briefly and clearly demonstrated that OMV immunization effectively protected ICR mice from the *A. baumannii* challenge, providing the necessary knowledge of a rational immunization procedure and actual protective efficacy of the OMV vaccine. In addition, the study particularly emphasized clarifying whether the OMV vaccine strategy remained effective in a pneumonia model, in which a more complete immunity including both systemic and mucosal responses might be required. The results demonstrated that immunization with OMVs provided significant protective effects against pulmonary infection with *A. baumannii*. Finally, based on an *in vitro* opsonophagocytic killing assay, the probable effective mechanisms and the protective potentials to clonally distinct clinical isolates were investigated, indicating that OMVs are a promising vaccine candidate for clinical application.

Theoretically, the intranasal administration of *A. baumannii* causes immune responses, such as cytokine production and immune cell recruitment to the lung, and, consequently, the invaded bacteria are cleared. Along with the clearance of bacteria, the inflammatory cell infiltration and the cytokine storm are eventually resolved. In the pneumonia model in the current study, a high bacterial burden in BALF, lung, and spleen was found one day after the *A. baumannii* challenge and reduced gradually from day 3 over the subsequent 14 days. Correspondingly, the accumulation of cytokines in BALF and the infiltration of inflammatory cells in the lung gradually reversed and completely resolved in 14 days. A previous study reported that lung infection with *A. baumannii* upregulated the production of cytokines in BALF, such as TNFα, IFN-γ, and IL-1β [10], which are important proinflammatory cytokines that are closely involved in innate immune responses. Our results confirmed these discoveries and, interestingly, also showed elevated levels of IL-33 and IL-13, two cytokines associated with Th2 responses. Recent studies indicated that IL-33 is a critical mediator of the innate immune response that is released by epithelial cells as an alarm upon pathogen invasion and promptly initiates the immune response. IL-33 may activate innate type 2 lymphoid cells and macrophages to express various cytokines, including IL-13 [33]–[35]. As is well known, IL-13 is a typical Th2 cytokine and plays a pivotal role in allergic airway inflammation. However, its role in *A. baumannii* infection is not clear, and its expression may indicate whether *A. baumannii* infection may be involved in the development or exacerbation of asthma. Therefore, it would be interesting to determine IL-13 expression in this study. The finding of elevated IL-33 levels indicated that IL-33 might play a role in the recruitment of immune cells to the lung and may promote bacterial clearance in the pneumonia model. This hypothesis is supported by a previous report in which IL-33 attenuates sepsis by enhancing neutrophil influx to the site of infection [33]. Furthermore, an increase in IL-13 levels was detected by ELISA in the current study, which is consistent with the results shown in a previous study [16].

Active immunization via the intramuscular route produced systemic IgG responses, which might contribute to the clearance of bacteria in body fluid and prevent further bacterial invasion into other tissues by mediating and promoting phagocytosis by the immune cells, such as macrophages and neutrophils. Interestingly, the intranasal challenge with *A. baumannii* following systemic immunization with OMVs rapidly elicited local antibody responses, including both IgA and IgG on the mucosal surface of the airway, which may provide additional benefits for enhancing the immediate bacterial clearance and preventing pulmonary infection. In addition, the presence of OMV-specific IgGs was also detectable in BALF in mice administered intravenous passive immunization with the antisera, most likely due to the transportation of IgG from the body fluid to the mucosal surface.

It has been reported that the intranasal administration of *A. baumannii* induces Th1 type responses and thus suppresses subsequent OVA-stimulated Th2 type airway allergic inflammation responses [36]. In the current study, IL-33 and IL-13 were found to be significantly elevated in the pneumonia model. These powerful factors promote Th2 type immune responses. To determine whether there is a type shift from the Th1 to Th2 responses, we also measured the IgG_1_ and IgG_2a_ levels at various time points after the OMV immunization and *A. baumannii* challenge, respectively. The results showed that OMV immunization elicited high levels of IgG_2a_, indicating strong Th1-like responses, and that pulmonary infection with *A. baumannii* maintained a similar IgG subtype profile. These findings were consistent with the strong expression of Th1 cytokines detected in BALF.

Our results showed that active immunization and passive immunization at day 1 post-challenge both significantly ameliorated the bacterial burden, inflammatory cell infiltration and cytokine accumulation in mice in pneumonia mode. The explanation might be that decreased bacterial loads caused by OMVs immunization consequently contributed to the reduced cytokine expression and immune cell infiltration. Overall, active immunization displayed stronger effects than passive immunization in this study. This finding may be explained by the higher, more persistent titers elicited by active immunization and the mucosal and humoral responses of antigen-specific antibodies. The results indicated that continuous administration of antisera for a few days may be needed to effectively control the infection, which also emphasized that the administration of the antisera suppressed the infection.

Although immunization herein appears to have lessened the severity of infection, a significant number of bacteria remain in the lungs, spleen and BALF. We noticed that in two previous studies involving a sepsis model, McConnell et al. reported that the IWC or OMV immunization reduced the bacterial loads in the spleen by approximately 10^4^ or 10^6^ times at 12 h after the infection compared with the control [8], [10]; however, there was no significant difference 12 h after the infection or only a difference of 10e2 times at 18 h after infection in a study reported by Fattahian et al. [15], in which the mice were immunized with a biofilm-associated protein (Bap) subunit and significantly protected from a lethal dose of *A. baumannii*. In the studies involving a pneumonia model, vaccination targeting a trimeric autotransporter protein Ata or polysaccharide PNAG produced bacterial loads in lung tissues that were only reduced approximately 2-5-fold or 3-5-fold depending on the strains used for the challenge [9], [11] The results are similar to that found in our current pneumonia model study, in which a 2-4-fold reduction was found at 24 h. The above results indicate that the differences of multiple factors, including the animal model (sepsis or pneumonia), animal species, strain virulence, and *A. baumannii* infection doses, and also perhaps the vaccine form, might have affected the fold reduction of bacteria loads and the actual final bacterial loads in tissues. Although only a 2-4-fold reduction of the bacterial load was found in the current study, in our opinion, OMV immunization has the potency to be clinically significant in pneumonia caused by *A. baumannii* infection. Our reasons may include the following: (1) It is important to note that the enhanced clearance of bacteria in a non-lethal animal model has not been shown to correlate with clinical benefits in humans, which indicates that it may not appropriate to conclude the amount of bacterial clearance in animals will prove effective in humans; (2) The limitation of a mouse model of *A. baumannii* infection is that mice clear a large portion of the inoculum even without vaccination, and this makes it quite challenging to observe significant differences in response to vaccination. Thus, we believe that the enhanced *in vivo* reduction in the bacterial loads, even only a 2-5-fold reduction, as shown in previous studies and in our current study, is an indicator of potential vaccination efficacy; (3) The data from the pneumonia model showed that vaccination cleared bacterial loads quicker than the controls during the experimental period of 5 days, and it will be expected that the effect of vaccination on bacterial clearance would be substantially more significant if the observation period of the experiment had been longer or if a more delicately designed experiment was performed. However, definite conclusions can only be drawn through human clinical studies.

The antisera mediated significant phagocytosis against six of seven clinical isolates of *A. baumannii*, which were collected separately from different clinical departments and hospitals showing different antibiotic-resistance spectrums. The results strongly indicated that OMV immunization provided broad protection against clonally distinct clinical isolates. However, it is worth noting that one isolate appeared to be resistant to the opsonic phagocytosis mediated by the antisera. Whether this isolate could be intervened efficiently *in vivo* requires further investigation. To some extent, this result emphasized that immunization with OMVs prepared from one strain maybe not always be sufficient to effectively protect against infection by other clinical isolates. Furthermore, considering the similar pattern of specifically reactive bands of these seven clinical isolates, it is still unclear whether the antibodies against the specific proteins that were prominent on the Western blot provided effective protection.

LPS is a potent immune-stimulatory molecule that can be found in high abundance in Gram-negative bacterial OMVs. The LPS content of the *A. baumannii* OMVs may have contributed to the amelioration of infection found in this study or cause vaccine toxicity in future applications. Our current study cannot completely exclude these possibilities. However, this study does provide a proof-of-concept for the potential applications of active immunization with OMVs or passive immunization with antisera to control *A. baumannii* infection, although the safety of these treatments needs to be further improved and assessed. Moreover, this study has also clearly shown that the antisera ameliorated infection and inflammation *in vivo* or mediated the killing activity of macrophages *in vitro*

In summary, by targeting specific populations such as hospitalized patients, long-term residents of healthcare facilities, and combat soldiers, immunological approaches may provide a viable alternative for preventing or treating medical issues caused by *A. baumannii* infections. OMVs are a potential vaccine candidate because they can be prepared rapidly and easily from the culture of isolates from a clinical outbreak. Furthermore, passive immunization with antibodies may provide an optional treatment approach in critically ill patients infected with multi- or pan-resistant bacterial strains.
